# Letter to the Editor on “Association of total testosterone levels with cardiometabolic diseases in men with erectile dysfunction”

**DOI:** 10.1093/sexmed/qfaf020

**Published:** 2025-04-06

**Authors:** Scott Selinger, Gregory R Thoreson

**Affiliations:** Department of Internal Medicine, University of Texas at Austin Dell Medical School, Austin, TX 78712, United States; Department of Surgery and Perioperative Care, University of Texas at Austin Dell Medical School, Austin, TX 78712, United States

We appreciate the work of Chen et al.^1^ in furthering the linkage between low testosterone and a myriad of comorbidities with increased cardiovascular disease. There are several clarifying points on their work we would like to add.

First, this information should serve to remind the medical and general community that there is little to no link between total testosterone and erectile dysfunction (ED), as ED severity was similar between men with low and normal testosterone and only a third of men presenting with ED had low testosterone. The lack of free testosterone levels is unfortunate, as it has been postulated that the symptom burden and disease risk correlate better with free than total levels.^2^ The inverse correlation of total testosterone and body mass index (BMI) is notable as BMI is inversely correlated with serum hormone binding globulin (SHBG), leading to higher free testosterone levels, while age is more directly associated with SHBG levels.^3^

Low testosterone can signal global cardiometabolic disease, as Chen noted nearly 40% increased incidence of diabetes, hypertension and dyslipidemia in the low testosterone group (though interestingly the average HbA1c was nearly the same, suggesting substantially worse diabetes control in the hypogonadal group). However, testosterone supplementation has no major effect on disease outcomes for these conditions including glycemic remission^4^ or cardiovascular disease outcomes.^5^ It may be that while testosterone is a marker for chronic disease and comorbidity, in the same vein as sodium, hemoglobin and vitamin D, it is not an effective target to reduce illness burden.

The strongest argument to be made by this longitudinal study is the potential for high impact interventions or touchpoints for men with multimorbidity presenting to urology clinics for erectile dysfunction. Long-heralded as the “canary in the coal mine” for cardiovascular disease, given the progressive and relative dearth of high quality accessible primary care combined with masculinity-associated dearth of timely healthcare-seeking behavior in men, those asking for help with ED have already opened the door to a discussion around lifestyle-based interventions to improve comorbid conditions and we as their trusted providers should make a point to walk through that door together.
